# Hexamethylene bisacetamide impairs NK cell-mediated clearance of acute T lymphoblastic leukemia cells and HIV-1-infected T cells that exit viral latency

**DOI:** 10.1038/s41598-019-40760-x

**Published:** 2019-03-13

**Authors:** Erica Giuliani, Maria Giovanna Desimio, Margherita Doria

**Affiliations:** Laboratory of Immunoinfectivology, Bambino Gesù Children’s Hospital, IRCCS, Piazza S. Onofrio 4, 00165 Rome, Italy

## Abstract

The hexamethylene bisacetamide (HMBA) anticancer drug was dismissed due to limited efficacy in leukemic patients but it may re-enter into the clinics in HIV-1 eradication strategies because of its recently disclosed capacity to reactivate latent virus. Here, we investigated the impact of HMBA on the cytotoxicity of natural killer (NK) cells against acute T lymphoblastic leukemia (T-ALL) cells or HIV-1-infected T cells that exit from latency. We show that in T-ALL cells HMBA upmodulated MICB and ULBP2 ligands for the NKG2D activating receptor. In a primary CD4^+^ T cell-based latency model, HMBA did not reactivate HIV-1, yet enhanced ULBP2 expression on cells harboring virus reactivated by prostratin (PRO). However, HMBA reduced the expression of NKG2D and its DAP10 adaptor in NK cells, hence impairing NKG2D-mediated cytotoxicity and DAP10-dependent response to IL-15 stimulation. Alongside, HMBA dampened killing of T-ALL targets by IL-15-activated NK cells and impaired NK cell-mediated clearance of PRO-reactivated HIV-1^+^ cells. Overall, our results demonstrate a dominant detrimental effect of HMBA on the NKG2D pathway that crucially controls NK cell-mediated killing of tumors and virus-infected cells, providing one possible explanation for poor clinical outcome in HMBA-treated cancer patients and raising concerns for future therapeutic application of this drug.

## Introduction

Natural killer (NK) cells are the major component of the innate immune system endowed with the capacity to recognize and kill virus-infected and transformed cells without prior antigen encounter. The function of NK cells is regulated by the net balance of opposite signals delivered by activating and inhibitory receptors that bind cognate ligands on the surface of target cells^1^. Upon viral infection or transformation, a cell usually loses human leukocyte antigen class I (HLA-I) molecules that function as ligands for inhibitory receptors and, simultaneously, upregulates ligands of activating receptors, hence becoming a target for NK cell-mediated lysis. The antitumor function of NK cells has been exploited in several clinical trials to treat cancer patients by means of NK cell adoptive transfer in either autologous or allogeneic settings^2–5^. However, therapeutic success can be affected by disease relapse in some patients, especially in children with acute lymphoblastic leukemia (ALL) or other hematological malignancies. This is why combination of NK cell-based immunotherapy with chemotherapeutic agents or other drugs that boost expression on tumor cells of ligands for NK-cell activating receptors is under investigation as novel anticancer strategy^6,7^.

Among NK cell activating receptors, the NK receptor group 2 member D (NKG2D) plays a key role in the recognition of both tumors and infected cells. In humans, NKG2D is expressed by all NK and CD8^+^ T cells and by subsets of γδ T cells, NKT cells, and CD4^+^ T cells, and recognizes several ligands (NKG2DLs): the major histocompatibility complex I-related chain A and B proteins (MICA and MICB) and UL16 binding protein 1–6 (ULBP1-6)^8^. Expression of NKG2DLs requires activation of NF-κB and other transcription factors, chromatin remodeling, and activation of the DNA Damage Response (DDR) pathway, henceforth is highly restricted in normal tissues but can be induced during viral infection and tumor transformation^9^. Ligand binding by NKG2D results in phosphorylation of a receptor-associated adaptor, DNAX-associated protein 10 (DAP10), followed by engagement and activation of the phosphatidylinositol 3-kinase (PI3-K) and downstream signaling molecules that potently stimulate NK cell-mediated elimination of virus-infected cells and tumors^10,11^. The importance of NKG2D-mediated response of NK cells against cancer has been demonstrated by immunogenetic, clinical and experimental studies^12^, also corroborated by the evidence that most anticancer drugs, including antimetabolic agents, antitumor antibiotics, and histone deacetylase inhibitors (HDACi), upmodulate NKG2DLs on tumor cells^7^. Moreover, NKG2D has a key role in infectious diseases, given that cells respond to most viral infection by upregulating NKG2DLs and, notwithstanding various mechanisms evolved by viruses to restrain NKG2DL cell-surface expression, they become targets for NKG2D-mediated recognition and killing by NK cells, as clearly demonstrated for HIV-1-infected CD4^+^ T cells^13^. Recently, we proposed that the NKG2D/NKG2DLs axis could be exploited to clear latent HIV-1 reservoirs persisting in infected patients despite suppressive antiretroviral therapy (ART), which represent a major drawback in the fight against HIV-1^14^. Actually, the ultimate ‘shock-and-kill’ approach to HIV-1 eradication implies administration of viral latency reversing agents (LRAs) that reactivate silent provirus, associated with the killing of cells harboring reactivated virus by the host immune system^15^. Candidate LRAs belong to different functional categories given that various mechanisms contribute to the establishment and maintenance of HIV-1 latency, including epigenetic silencing of the viral promoter and sequestration in inactive complexes of factors required for viral transcription initiation (*i.e*. NF-κB) and elongation (*i.e*. positive transcription elongator factor b, P-TEFb)^16,17^. To date, clinical trials in which LRAs have been administered to ART patients demonstrated modest or no clearance of HIV-1 reservoirs despite reactivation of viral gene expression, hence adjuvant immune-based strategies are being evaluated^18–20^. Of note, most LRAs under investigation have been used in tumor immunology studies and clinical trials for non HIV-1-related diseases because of their capacity to upregulate NKG2DLs^9,21^, indicating that latent HIV-1 and NKG2DLs are under the control of common regulatory pathways. As a matter of fact, we recently showed that two candidate LRAs with HDACi or protein kinase C agonist (PKCa) activity (*i.e*. SAHA and prostratin -PRO-, respectively) can simultaneously induce/derepress latent HIV-1 and NKG2DLs^14,22^. In addition, we and others have shown that IL-15 potently boosted NKG2D expression and cytotoxicity of NK cells against T cells that exit viral latency, suggesting that combining LRA administration and immunotherapy with IL-15-activated NK cells represents a promising approach towards HIV-1 eradication^14,23^.

Aside their capacity to upmodulate NKG2DLs, candidate drugs with anticancer and/or LRA properties should be carefully tested for their direct effect on immune effector cells. Indeed, accumulating evidence suggests that some therapeutic agents, particularly HDACis, induce the downregulation of NKG2D and other activating receptors on NK cells and, more in general, can decrease NK or CD8^+^ T cell viability and/or cytotoxicity against tumors or infected target cells^24–29^.

In the present study we thoroughly investigated the impact on the NKG2D/NKG2DL axis of hexamethylene bisacetamide (HMBA), a prototype hybrid bipolar compound with both anticancer and LRA features. HMBA was initially developed as an anticancer drug based upon its ability to induce differentiation and growth arrest of various transformed cells, including promyelocytic leukemia, breast cancer, colon carcinoma, and melanoma cells^30^. In transformed cells, HMBA leads to cell cycle arrest in G_0_/G_1_, loss of proliferative capacity, and also, in some tumor cell lineages, apoptotic cell death^31^. These events have been associated with HMBA-induced changes affecting proteins that regulate the cell cycle (*e.g*. pRB, p53, E2F), anti-apoptotic factors (*e.g*. Bcl-2, Notch1), and PKC^32–35^. On the basis of *in vitro* evidences, HMBA has entered into clinical trials for leukemia and myelodysplastic syndrome^36,37^. However, the lack of significant hematologic improvement in HMBA-treated patients has hampered further testing of this drug in oncologic trials. The interest in HMBA was recently renewed because of its reported capacity to reactivate latent HIV-1 via activation of P-TEFb and elongation of viral transcripts^38,39^. Specifically, HMBA was shown to disrupt the inactive complex in which P-TEFb is sequestered through activation of Ca^2+^ signaling and of PP2B and PP1α phosphatases^40^, as well as *via* PI3-K stimulation^38^. HMBA can also stimulate initiation of HIV-1 transcription, albeit with a modest effect^41^. Thus, the use of HMBA in combination with PKCa such as PRO, which are known to activate NF-κB signaling pathway, is under investigation as a potential strategy to reduce the size of latent HIV-1 reservoir^42,43^.

Here we assessed the effects of HMBA on NKG2DL expression in both acute T lymphoblastic leukemia (T-ALL) cells and in HIV-1-infected CD4^+^ T cells that exit from viral latency, as well as on the phenotype and function of NK cells. We show that, notwithstanding a stimulatory effect on NKG2DL expression on target cells, HMBA downmodulates NKG2D in NK cells, hence inhibiting their cytotoxicity against T-ALL cells and T cells harboring reactivated HIV-1. Moreover, NK cells exposed to HMBA display low DAP10 levels and do not respond to IL-15 stimulation. These results indicate that HMBA can exert a detrimental effect on NK cells in the context of cancer therapies or ‘shock-and-kill’ approach to HIV-1 eradication.

## Results

### HMBA upmodulates NKG2DLs on T-ALL cells

**Figure 1:**
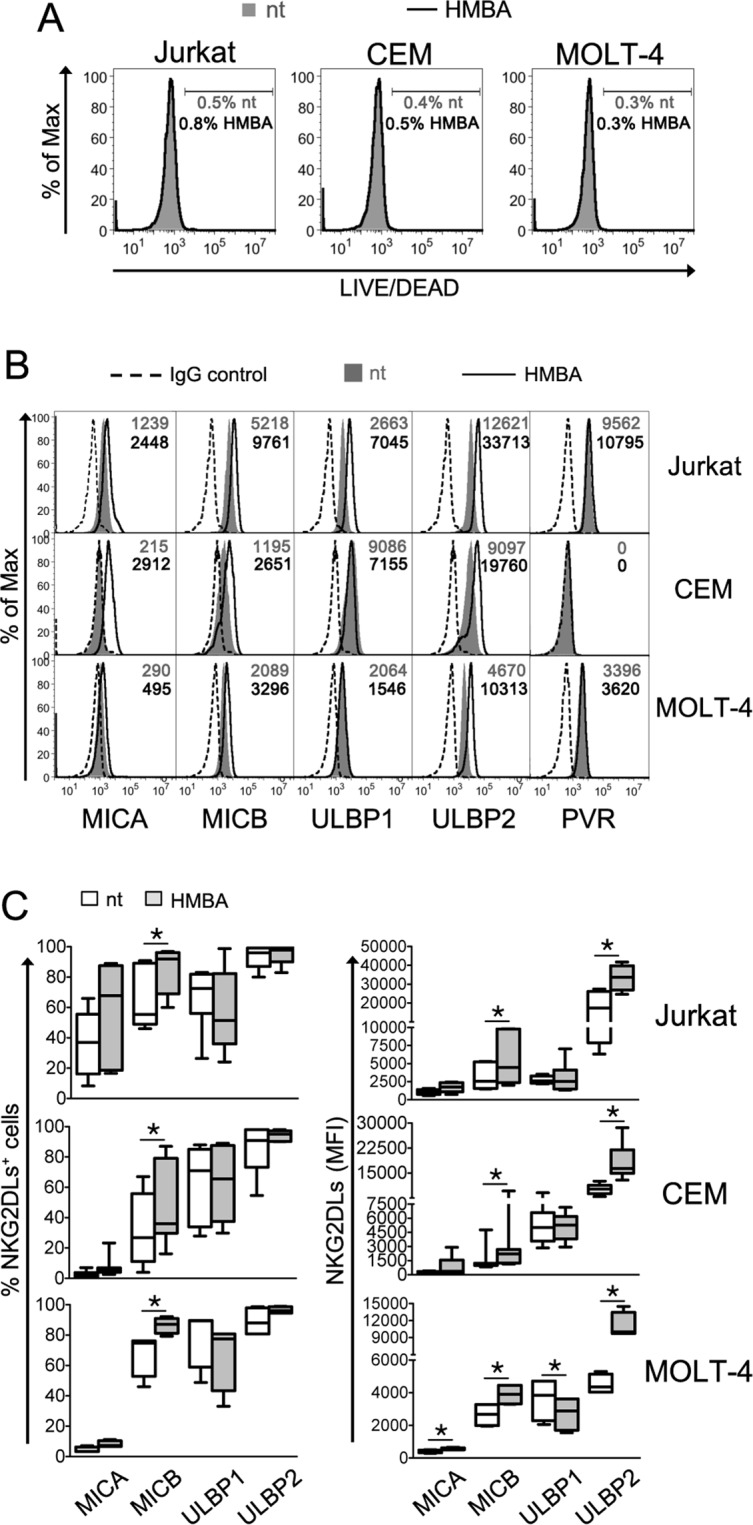
HMBA induces NKG2DL expression on leukemia T cells. Three T lymphoblastoid cell lines, Jurkat, CEM and MOLT-4, were cultivated for 18 h in medium alone (not treated, nt) or in the presence of 5 mM HMBA and then analyzed by flow cytometry to evaluate cell viability and measure the cell-surface expression of NKG2DLs and PVR. (**A**) The percentage of DEAD^+^ cells among nt (filled grey histograms, grey percentages) and treated cells (open histograms, black percentages) is shown. (**B**) Histograms show the fluorescence distribution of cells labeled with mAbs against MICA, MICB, ULBP1, ULBP2, and PVR in a representative experiment. Dashed line, filled gray histogram, and solid line represent staining with isotype control IgG, nt, and HMBA-treated cells, respectively. The MFI values for nt cells (gray) and HMBA-treated cells (black) are indicated. (**C**) NKG2DL expression, both % of positive cells and MFI, was determined as shown in panel A in 3 independent experiments. Whiskers box analysis shows median (bar), 25th–75th percentile (box), and minimum and maximum (vertical lines) values. *P < 0.05 by Wilcoxon matched-pairs test.

To investigate the impact of HMBA on NKG2DL expression in tumor cells, we selected three cell lines derived from patients with acute T lymphoblastic leukemia (T-ALL): Jurkat, CEM, and MOLT-4. After 18 h culture with or without the addition of HMBA at a 5 mM concentration that is not toxic and optimal for inducing differentiation of transformed cells^44^, cells were analyzed by FACS for the surface expression of four NKG2DLs (MICA, MICB, ULBP1, and ULBP2) as well as of PVR (CD155), a nectin-like molecule that is frequently upregulated in tumors and functions as ligand for another NK-cell activating receptor, DNAM-1^45^. As expected, we did not observe losses in cell viability upon HMBA treatment (Fig. 1A). With the exception of MICA that was barely detectable in CEM and MOLT-4 cells, all tested NKG2DLs were expressed in the three cell lines (Fig. 1B), consistent with the presence of at least one of these molecules on leukemia cells of most patients^46,47^. Upon HMBA exposure, the three cell lines demonstrated a significant ~2-fold increase in MICB and ULBP2 expression levels, while MICA was only minimally augmented and ULBP1 was either not affected or, only in MOLT-4 cells, slightly decreased (Fig. 1B,C). Moreover, the basal expression of PVR, which is elevated in Jurkat and MOLT-4 cells and absent in CEM cells, did not change following HMBA treatment (Fig. 1B). Thus, although restricted to MICB and ULBP2, the upmodulation of NKG2DL by HMBA has the potential to enhance T-ALL cells killing via NKG2D^+^ cells.

### Impact of HMBA on NK cell phenotype

**Figure 2:**
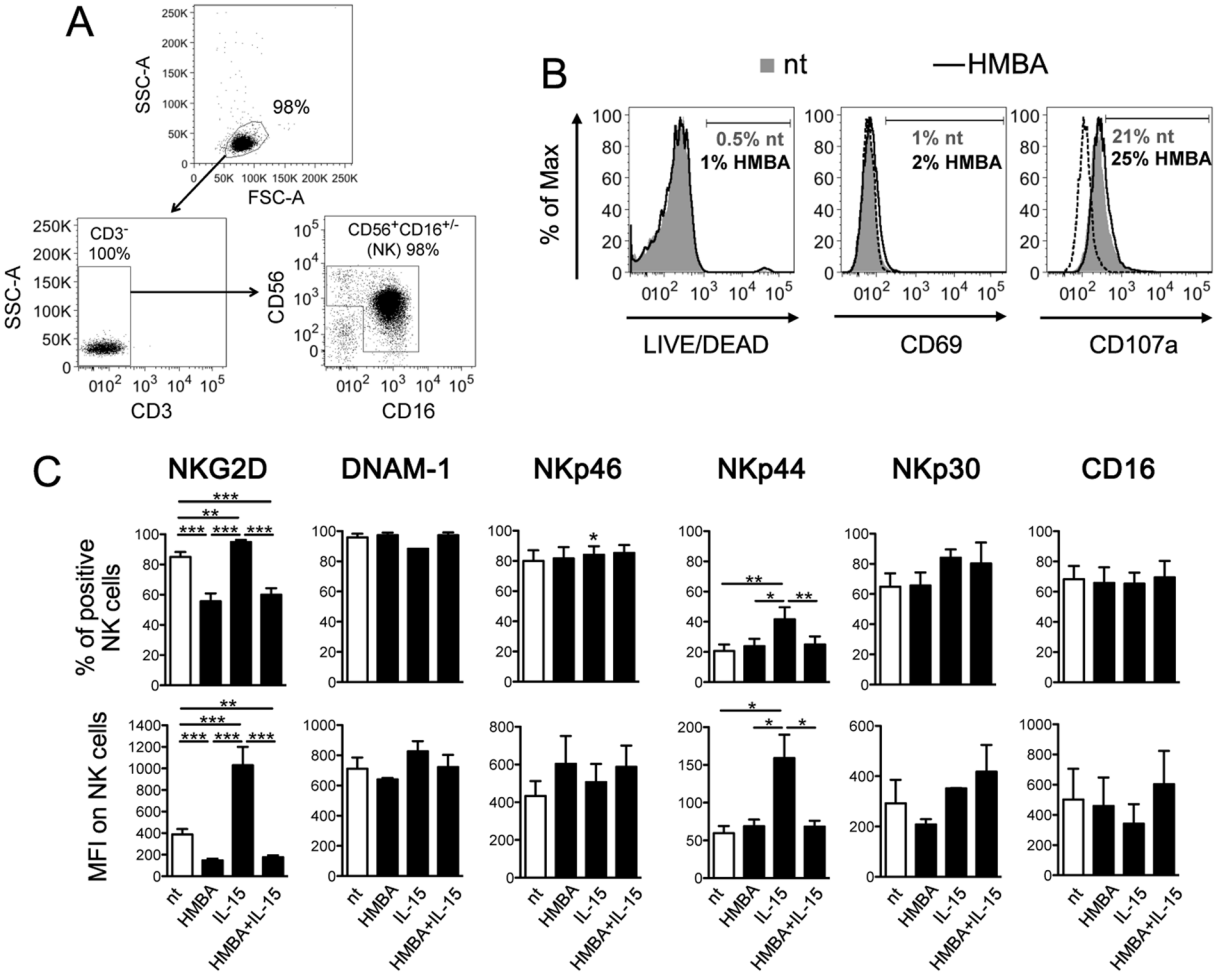
HMBA downmodulates NKG2D on NK cells. (**A**) The purity of NK cells isolated from PBMCs was examined by flow cytometry measuring the frequency of CD3^−^CD56^+^CD16^+/−^ cells. (**B**,**C**) NK cells were cultured for 18 h in medium alone (nt) or supplemented with 5 mM HMBA and examined for the expression of various markers: (**B**) the percentage of DEAD^+^, CD69^+^ and CD107a^+^ NK cells among nt (filled grey histograms, grey percentages) and treated cells (open histograms, black percentages) is shown together with control IgG signal (dashed line) for a representative experiment; (**C**) both the % of positive cells and MFI for NKG2D, DNAM-1, NKp46, NKp44, NKp30, and CD16 are shown for NK cells nt and treated with HMBA and/or 12.5 ng/ml of IL-15. Experiments were performed with at least 5 independent donors. Bars represent mean ± SEM. *P < 0.05, **P < 0.01, ***P < 0.001 by two-tailed paired *t* test.

Next, we examined the effects of HMBA on NK cells. First, primary NK cells isolated from healthy donors were cultivated for 18 h with or without 5 mM HMBA, then analyzed by flow cytometry. We found that HMBA did not affect NK cell viability and had no effect on the expression of the CD69 activation marker or on CD107a that measures degranulation activity (Fig. 2A,B). These results are in line with a previous report showing that markers of cell activation (*i.e*. CD69, CD25, CD38, HLA-DR) or proliferation (*i.e*. Ki67) did not change in PBMCs upon treatment with HMBA^48^. We then assessed the expression of six activating receptors that regulate NK cell cytotoxic responses, including NKG2D, DNAM-1, the NKp46, NKp44, and NKp30 natural cytotoxicity receptors (NCRs), and the CD16 (FcgRIII) Fc receptor that mediate antibody-dependent cellular cytotoxicity (ADCC). This analysis was performed following 18 h culture with or without HMBA in the presence or absence of IL-15, a physiologic stimulus of NK cell activity. Exposure to HMBA alone caused a significant decrease in the expression of NKG2D in terms of frequency of NKG2D^+^ cells and NKG2D MFI, while other receptors were not affected (Fig. 2C). As expected, NK cells responded to short-term IL-15 stimulation by upregulating both NKG2D and NKp44^14,22^, but not in the presence of HMBA. Thus, in NK cells HMBA suppresses cell-surface NKG2D expression and abrogates both NKG2D and NKp44 upmodulation induced by IL-15.

### NKG2D downregulation by HMBA

**Figure 3:**
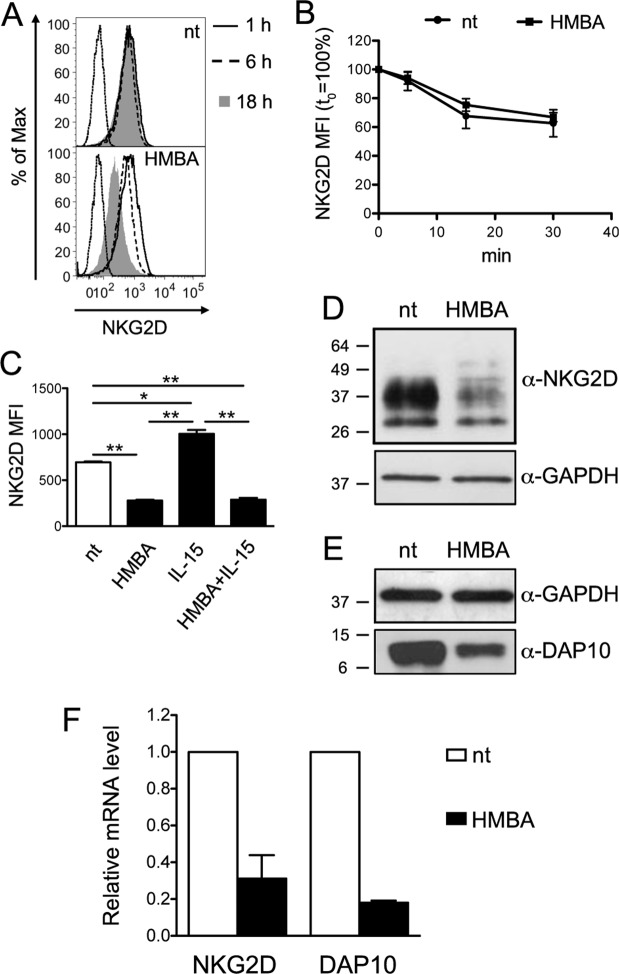
HMBA affects NKG2D and DAP10 expression. (**A**) Cell-surface NKG2D expression on NK cells at various time points following HMBA treatment. Histograms show NK cells treated or not (nt) with 5 mM HMBA for 1 h (solid line), 6 h (dashed line) and 18 h (filled grey histograms) in one representative experiment out of three. (**B**) Kinetics of NKG2D internalization in NK cells treated or not with HMBA for 18 h. Mean ± SEM values of NKG2D MFI were calculated over time with respect to initial expression (set to 100%) in 3 independent experiments. (**C**) Intracellular NKG2D expression was measured in permeabilized NK cells following 18 h treatment with HMBA and 12.5 ng/ml of IL-15, either alone or in combination, as compared to nt cells. NKG2D MFI (mean ± SEM) in 3 independent experiments is shown. *P < 0.05, **P < 0.01 by two-tailed paired *t* test. (**D**,**E**) Equal amounts of total cellular lysate of NK cells treated or not with HMBA for 18 h were analyzed by western blotting with anti-NKG2D (**D**) or anti-DAP10 (**E**) antibodies as well as with anti-GAPDH mAb to confirm equal protein loading. Molecular mass standards (kDa) are indicated. One representative of four independent experiments is shown. Uncropped blots are presented in Supplementary Fig. S1. (**F**) Relative NKG2D and DAP10 mRNA levels in NK cells treated with HMBA compared with control nt cells was measured by Real-time qPCR. Shown are mean ± SEM from three independent experiments performed in triplicate.

To gain insights into NKG2D downregulation by HMBA, we analyzed NKG2D turnover at the NK cell membrane, which is the major mechanism through which expression of this receptor is regulated^49^. By analyzing various time points, we found that NKG2D downmodulation by HMBA was a slow phenomenon, starting around 6 h post-treatment and reaching a maximum after 18 h (Fig. 3A). Next, we performed a FACS-based endocytosis assay triggered by anti-NKG2D antibody, showing that the rate of NKG2D internalization was maintained in NK cells treated for 18 h with HMBA if compared to untreated cells (Fig. 3B). Moreover, by immunolabeling intracellular NKG2D in NK cells treated with HMBA and IL-15, either alone or in combination, we found that the extent of NKG2D expression within HMBA-treated cells reflected that observed at the plasma membrane (Fig. 2C), with a 2-fold reduction that occurred with or without simultaneous addition of IL-15 that, when used alone, increased intracellular NKG2D amounts (Fig. 3C).

Finally, NKG2D expression was analyzed at both protein and mRNA levels. By western blotting analysis of total lysates of NK cells, we found that overall protein amounts of NKG2D and its DAP10 adaptor were strongly reduced by HMBA (median 75% and 60% reduction, respectively; Fig. 3D,E show representative blots). Of note, this effect of HMBA was associated with a dramatic decrease of NKG2D and DAP10 mRNA levels (median 70% and 80% reduction, respectively; Fig. 3F).

Overall, these data indicate that NKG2D downregulation by HMBA does not result from accelerated receptor internalization or retention in intracellular compartments, but is rather due to impaired gene expression and, hence, neo-synthesis of NKG2D. Furthermore, results indicate that HMBA also suppresses the expression of DAP10 that is required for stable NKG2D anchorage at the cell membrane^50,51^.

### HMBA decreases NK cell cytotoxicity

**Figure 4:**
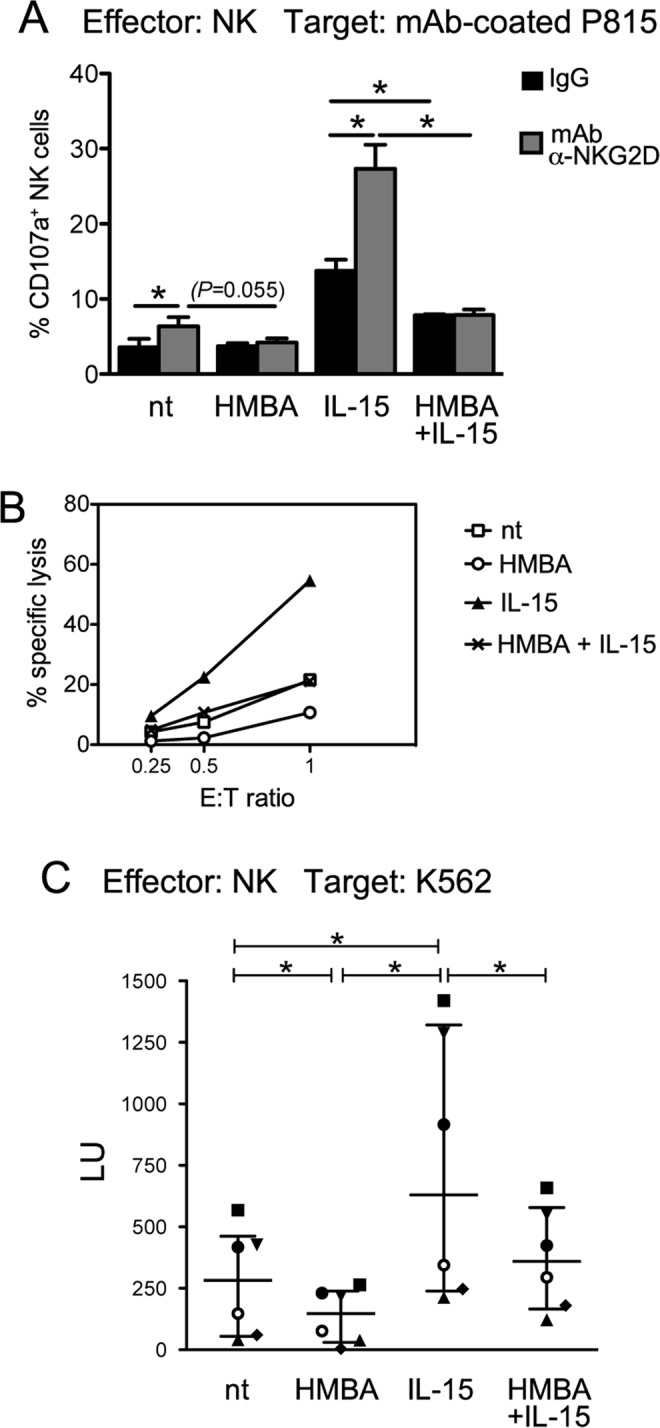
Impact of HMBA on NK-cell cytotoxicity. (**A**) A redirected antibody-dependent degranulation assay was performed with PBMCs cultivated for 18 h in medium alone (nt) or in the presence of 5 mM HMBA and/or 12.5 ng/ml IL-15. PBMCs were incubated for 6 h with P815 cells loaded with anti-NKG2D (α-NKG2D) mAb or control IgG in a re-directed degranulation assay. Then, cells were stained and analyzed by flow cytometry for the expression of CD107a on gated NK cells. The mean ± SEM percentage of CD107a^+^ NK cells measured in 3 independent experiments is shown. *P < 0.05 by two-tailed paired *t* test. (**B**,**C**) NK cells cultivated for 18 h without stimuli (nt), with HMBA and/or IL-15 were tested for cytotoxicity against K562 cell targets at different E:T ratio. (**B**) The percent specific lysis of a representative experiment out of 6 is shown. (**C**) The NK cell-mediated lysis measured in 6 independent experiments was converted in lytic units (LU, median and interquartile range). Each symbol represents 1 donor. *P < 0.05 by Wilcoxon matched-pairs test.

The negative impact on NKG2D expression suggests that HMBA may have deleterious effects on NK cell cytotoxic responses. To gain insights into this aspect, we set up a redirected killing assay to measure granule exocytosis (*i.e*. CD107a expression) by purified primary NK cells treated or not with HMBA and/or IL-15 upon interaction with P815 cells coated with anti-NKG2D mAb or, as control, mouse IgG_1_. In these settings, antibody-mediated triggering of NKG2D resulted in a ~2-fold increased frequency of CD107a^+^ cells among both untreated and IL-15-stimulated NK cells, whereas this increase was completely abrogated by exposure to HMBA (Fig. 4A). Interestingly, also IL-15-induced increment of basal % of CD107a^+^ NK cells incubated with IgG-coated P815 cells was significantly reduced if NK cells had been simultaneously exposed to HMBA. Moreover, to measure their killing activity, treated NK cells were challenged with K562 cells, a NKG2DL^+^ HLA-I-devoid erythroleukemia cell line that serves as established target of NK cytotoxicity. To this end, NK cells derived from 6 donors were pretreated or not with HMBA and/or IL-15, then used as effectors at various effector-to-target (E:T) ratio in a FACS-based assay that measures the percentage of specific lysis. Figure 4B,C shows that exposure to HMBA impairs the constitutive capacity of NK cells to kill K562 targets and, in addition, inhibits the enhancement of NK-cell cytotoxicity by IL-15. In sum, results demonstrate a detrimental effect of HMBA on NKG2D-mediated activation and, more in general, on IL-15-induced cytotoxicity of NK cells.

### Lysis of leukemia T cells by NK cells is inhibited by HMBA

**Figure 5:**
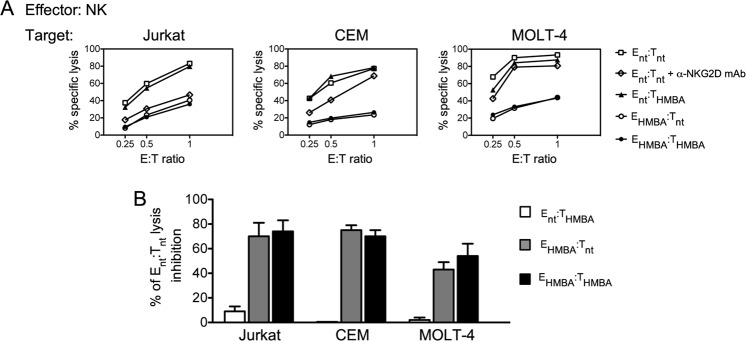
HMBA inhibits NK cell-mediated killing of leukemia T cells. (**A**,**B**) NK cells either treated with HMBA (E_HMBA_) or not treated (E_nt_) were used as effectors at various E:T ratio in a 4 h cytotoxicity assay against Jurkat, CEM and MOLT-4 target cells that have been as well exposed (T_HMBA_) or not to HMBA (T_nt_). (**A**) The percent specific lysis of a representative experiment out of 3 is shown. Results obtained with untreated NK cells (E_nt_) pre-incubated with saturating amounts of anti-NKG2D blocking mAb (α-NKG2D) and tested against T_nt_ are also shown. (**B**) Data are expressed as specific lysis inhibition at E:T ratio of 1:1 relative to E_nt_:T_nt_ lysis set at 100% (mean ± SEM, n = 3).

Altogether, results suggest that HMBA can have opposite effects on the NKG2D/NKG2DL axis as the drug, on one hand, upmodulates NKG2DLs on target cells and, on the other hand, reduces NKG2D expression and cytotoxicity in NK cells. Then, we investigated the overall impact in NK cell-mediated killing of T-ALL cells when both effectors and targets are exposed to HMBA. To this end, target cells (Jurkat, CEM, and MOLT-4) and effector NK cells were cultivated for 18 h in medium alone (referred as T_nt_ and E_nt_, respectively) or supplemented with HMBA (T_HMBA_ and E_HMBA_, respectively) prior being used in lysis assays at various E:T ratio. In pilot experiments we observed that, when not stimulated by cytokines, purified primary NK cells displayed very low to null killing activity against T-ALL cells (data not shown), thus all NK cell cultures were supplemented with IL-15. In agreements with previous reports^52,53^, lysis of untreated Jurkat, CEM, and MOLT-4 cells by NK cells was NKG2D-dependent, since it was significantly reduced by masking the receptor with specific mAb (51 ± 10%, 36 ± 8%, and 32 ± 7%, respectively, mean ± SEM lysis inhibition calculated at three E:T ratio in 3 independent experiments; a representative experiment is shown in Fig. 5A). Unexpectedly, we found that treatment of the three cell targets with HMBA did not increase their susceptibility to killing by untreated effectors (Fig. 5A shows a representative set of results). In addition, alongside with the negative effects in NK cells, prior HMBA exposure of NK cells resulted in a strong inhibition of their capacity to kill untreated targets, that is, by comparing E_HMBA_:T_nt_ vs E_nt_:T_nt_, lysis was inhibited by 70 ± 11%, 75 ± 4%, and 43 ± 6% for Jurkat, CEM, and MOLT-4 cells, respectively (Fig. 5A,B). A similar inhibition was also observed when both effectors and targets have been treated with HMBA (Fig. 5A,B). Therefore, MICB and ULBP2 upmodulation by HMBA on T-ALL cells (Fig. 1B,C) does not enhance their susceptibility to NK-cell killing and, moreover, HMBA impairs the overall cytotoxicity of IL-15-stimulated NK cells against T-ALL targets, possibly affecting other activating pathways in addition to the one mediated by NKG2D.

### HMBA synergizes with PRO at upregulating ULBP2 on HIV-1-infected CD4+ T cells that exit from viral latency

**Figure 6:**
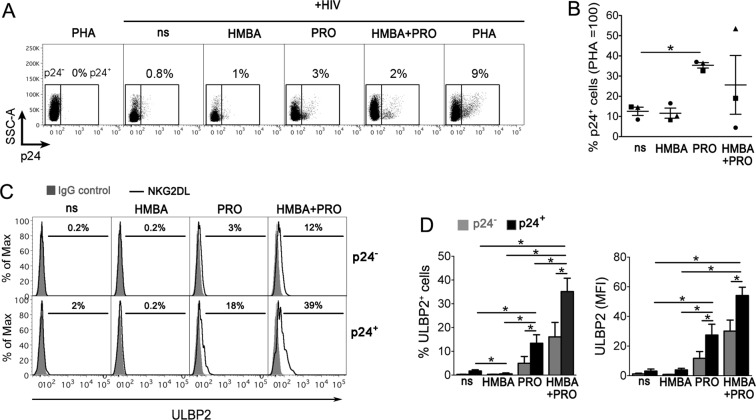
HMBA synergizes with PRO at upregulating ULBP2 on HIV-1-infected T cells that exit from viral latency. (**A**–**D**) To establish viral latency, freshly isolated CD4^+^ T cells were cultivated in the presence of CCL19 for 1–3 days, infected or not with HIV-1, and further cultured for 3 days, as described in details in Materials and Methods. Then, cells were treated with 5 mM HMBA or 1 μM PRO, alone or in combination, stimulated with 10 μg/ml PHA or not stimulated (ns), further cultivated for 3 days and finally analyzed by 2-color flow cytometry for the expression of intracellular p24 and cell-surface ULBP2. (**A**) Representative dot plots show the frequency of reactivated p24^+^ cells gated by setting non-infected PHA-stimulated control cells at 0%. (**B**) Percentage of p24^+^ cells was determined as shown in panel A in three independent experiments and normalized to HIV-infected PHA-treated cultures (mean ± SEM). Each symbol represents 1 donor. (**C**) Histograms show ULBP2 fluorescence on gated p24^−^ (top panels) and p24^+^ (bottom panels) cell populations measured in a representative experiment on ns, HMBA-, PRO-, and HMBA + PRO-stimulated cell samples. Signals obtained with control IgG (filled histograms) and the percentage of ligand-positive cells are shown. (**D**) ULBP2 expression (mean ± SEM), both % of positive cells and MFI, was determined in p24^−^ (grey bars) and p24^+^ (black bars) cells as shown in panel C in 3 independent experiments. *P < 0.05 by two-tailed paired *t* test.

We recently showed that in CD4^+^ T cells latently infected with HIV-1, the virus upmodulate NKG2DLs, particularly ULBP2, when reactivated by signaling modulators such as phytohemagglutinin (PHA) and PRO, or by SAHA, a polar compound with HDACi activity that is structurally related to HMBA^14,22^. As HMBA stands among candidate LRAs to be employed in HIV-1 eradication strategies, we investigated whether HMBA could modulate NKG2DLs in the context of viral reactivation and, hence, favor NKG2D-mediated recognition of cells that exit viral latency. Previously reported data indicate that, while the LRA activity of HMBA is evident in latently HIV-1-infected T cell lines^38,42,54^, HMBA may not be effective at stimulating latent HIV-1 in primary CD4^+^ T cells derived from ART patients if not occasionally^39,42,48,55^, unless used in combination with another LRA such as PRO^42^. Therefore, we established latent HIV-1 infection in primary resting CD4^+^ T cells derived from healthy donors and cultivated these cells for 72 h in medium alone (not stimulated, ns) or supplemented with 5 mM HMBA and/or 1 μM PRO, or, as maximal stimulation, with 10 μg/ml of phytohemagglutinin (PHA), then analyzed the expression of intracellular p24 (HIV-1 Gag capsid antigen) and cell-surface ULBP2 levels (representative and summary data with 3 donors are shown in Fig. 6A–D). We found that individual treatment with PRO but not HMBA induced the appearance of p24^+^ cells above the levels due to endogenous viral reactivation in ns cultures (35 ± 1% vs 12 ± 2%, *P* = 0.018, fixing p24^+^ cells in PHA samples at 100%; Fig. 6A,B), associated with an induction of ULBP2 on p24^+^ cells both as %ULBP2^+^ cells and ULBP2 MFI (Fig. 6C,D). We also found that, if compared with PRO alone, the simultaneous exposure to HMBA and PRO reactivated HIV-1 to a higher extent in 1 donor and, conversely, to a lower extent in 2 donors (35 ± 1% vs 26 ± 15% p24^+^ cells, mean ± SEM, *P* = 0.572), nonetheless ULBP2 expression on p24^+^ cells was further upmodulated to significantly higher levels in all tested donors (Fig. 6C,D). Apparently, HMBA has a poor latency reversal activity, at least in a primary CD4^+^ T cell-based model system that measures accumulation of a late viral protein. However, when used in association with PRO, HMBA can further enhance ULBP2 expression induced by PRO-reactivated HIV-1.

### NK cell-mediated clearance of autologous CD4+ T cells with reactivated HIV-1 is impaired by HMBA

**Figure 7:**
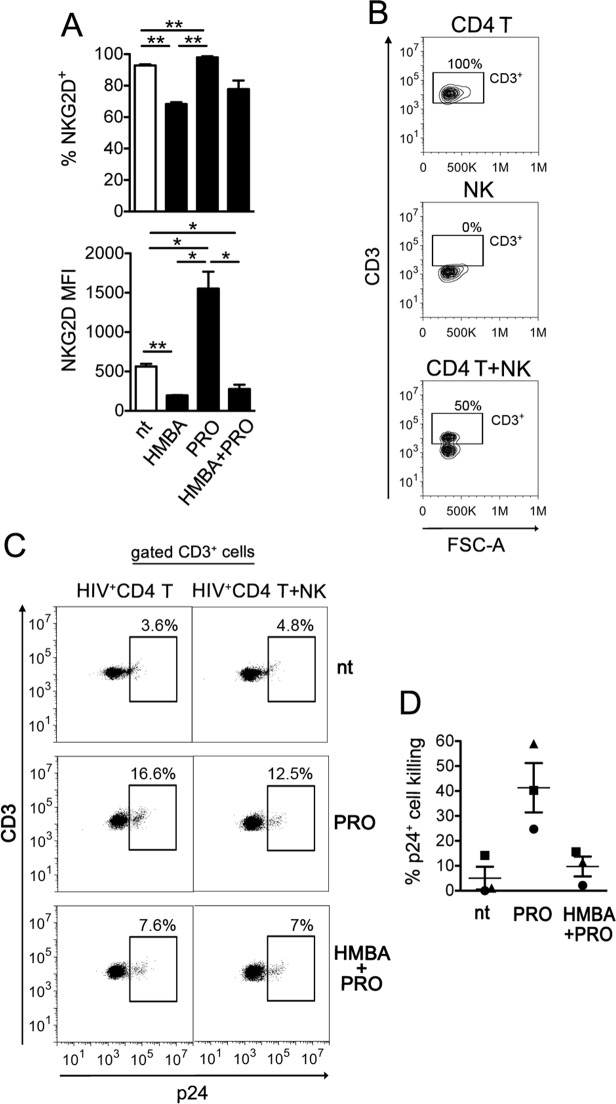
HMBA impairs the capacity of NK cells to suppress latently HIV-infected CD4^+^ T cells harboring PRO-reactivated virus. (**A**) The expression of NKG2D on NK cells not treated or exposed to HMBA, PRO, or HMBA + PRO for 18 h was measured as % of positive cells and NKG2D MFI (mean ± SEM, n = 3). (**B**–**D**) A co-culture assay of latently infected CD4^+^ T cells at day 2 post-stimulation with 1 μM PRO alone or in combination with 5 mM HMBA and autologous NK cells at a 1:1 E:T ratio was performed overnight (18 h) in the presence of the same drug(s). Then, cells were analyzed to measure the frequency of p24^+^ cells among gated CD3^+^ targets and calculate % reduction of 24^+^ targets by NK cells. (**B**) The CD3^+^ cell gate was set on cultures of targets alone, so that effectors cells were excluded, as shown in control cultures of effectors alone as well as in co-cultures of targets and effectors in a representative experiment. (**C**) A representative set of results for nt, PRO-, and HMBA + PRO-exposed cultures is shown. (**D**) The NK cell mediated clearance of p24^+^ cells was measured in 3 independent experiments and expressed as % 24^+^ killing (mean ± SEM) as described in Materials and Methods. Each symbol represents 1 donor. *P < 0.05, **P < 0.01 by two-tailed paired *t* test.

Finally, we investigated the overall impact in NK cell-mediated killing of latently infected CD4^+^ T cells when both effector and target cells are exposed to PRO and HMBA. First, the expression of NKG2D on treated NK cells was analyzed, showing that the receptor was upmodulated by PRO, in line with our previous report^22^, but this effect was abrogated in the presence of HMBA resulting in NKG2D downregulation in HMBA as well as in HMBA + PRO treated cells as compared with nt cells (Fig. 7A). Next, freshly isolated resting CD4^+^ T cells were infected with HIV-1 to establish viral latency and treated or not with PRO or HMBA + PRO as described above. After 54 hr of culture, CD4^+^ T cells were collected, resuspended in the same treatment condition (nt, PRO, or HMBA + PRO) either alone or together with autologous NK cells purified from a cryopreserved aliquot of PBMCs of the same donor at an E:T ratio of 1:1, and cultivated for further 18 h. At last, the frequency of p24^+^ cells was measured by flow cytometry within target CD3^+^ cell population (Fig. 7B,C). By comparing CD4^+^ T with CD4^+^ T/NK cultures of 3 donors, we found that the frequency of p24^+^ cells was consistently reduced by the presence of NK cells in PRO cultures (41 ± 10%) as we previously reported^22^, but not in nt or HMBA + PRO cultures (5 ± 5% and 10 ± 4%, respectively) (Fig. 7C,D). Therefore, when both effectors and targets are treated, the inclusion of HMBA can suppress the NK cell-mediated clearance of the HIV-1 reservoir in CD4^+^ T cells reactivated by PRO.

## Discussion

HMBA belongs to a class of hybrid polar compounds with potential anti-cancer activity that has been used to treat myelodysplastic syndrome and acute leukemia, but later dismissed on the basis of limited efficacy^36,37^. Recent data disclosing HMBA capacity to reactivate latent HIV-1 reservoirs has opened the possibility that the drug re-enters into the clinics in the context of HIV-1 eradication strategies^38,39,42,48,55^. Herein, we demonstrated that HMBA has opposite dual effects on the NKG2D/NKG2DLs axis that crucially controls NK cell mediated elimination of tumors and virus-infected cells. Indeed, we provided the first evidence that HMBA upmodulates NKG2DLs while, on the other hand, impairs NKG2D-mediated responses of NK cells, which has important implications for HMBA therapeutic application.

We showed that, in treated Jurkat, CEM, and MOLT-4 cells, HMBA specifically increased the cell-surface expression of MICB and ULBP2 without affecting MICA or ULBP1. This effect is analogous to that of other anti-cancer drugs, including the natural occurring polyphenol Resveratrol, various HDACi (*e.g*. SAHA, Romidepsin, Trichostatin A, Valproate), and proteasome inhibitors (*e.g*. Bortezomib), that were shown to upregulate MICA/B (detected with an antibody recognizing both MICA and MICB molecules) and ULBP2 in Jurkat or MOLT-4 cells^56–60^. HMBA may possibly upregulate MICB and ULBP2 at the transcriptional level by its reported capacity to activate E2F and p53^33,35^, which positively regulate expression of *NKG2DL* genes^9^. In addition, it is feasible that P-TEFb activation by HMBA can enhance MICB and ULBP2 mRNA elongation, a mechanism through which cells bypass transcriptional pausing of several genes belonging to the innate immune system to allow rapid and robust immune responses^61^. Unexpectedly, we found that MICB and ULBP2 upregulation by HMBA in T-ALL cells was not associated with increased susceptibility to NK-cell mediated killing (Fig. 5A). These results are in contrast with the fact that NK cells kill leukemic T cells in a NKG2D-dependent fashion (this study and ref.^52^), and with a higher efficiency when MICA/B and/or ULBP2 are induced by HDACi or other drugs^56–59^. We hypothesize that enhanced susceptibility of T-ALL cells to NK-cell killing is strictly correlated with the surface density of a NKG2DL distinct from those induced by HMBA such as MICA. Indeed, by using a specific anti-MICA antibody, treatment of Jurkat or a Jurkat-derived cell line (J-LatGFP) with SAHA or other HDACi resulted in a 4-fold increase of MICA expression along with MICB and ULBP2 upmodulation (ref.^14^ and our unpublished data). Apparently, optimal exposure of T-ALL cells to NKG2D-mediated responses of NK cells may require induction of MICA and/or other NKG2DL that cannot be achieved with HMBA, but rather with HDACis.

Another unexpected result of the present study consists in the negative impact of HMBA on NK cell phenotype and function. We found that, in NK cells, HMBA reduced NKG2D mRNA levels and overall protein amounts, resulting in cell-surface NKG2D downmodulation and decreased degranulation activity upon NKG2D triggering in a redirected killing assay. Moreover, the capacity of HMBA-treated NK cells to kill tumor targets that largely depend on NKG2D/NKG2DL interactions, such as T-ALL or K562 cells, was drastically reduced. HMBA also reduced the NKG2D adaptor DAP10 at both mRNA and protein level, a phenomenon that can account not only for impaired signaling of cell-surface NKG2D molecules, but also for receptor downregulation. Accumulating evidence, indeed, shows that association with DAP10 is required for NKG2D protection from degradation and stable expression at the cell membrane^50,51^. Further studies are required to determine the precise mechanism by which HMBA reduces NKG2D and DAP10 gene transcription. Importantly, by modulating DAP10 amounts, cytokines regulate NKG2D expression levels and, more in general, the effector function of NK cells, with a positive effect on DAP10 protein synthesis exerted by cytokines belonging to the common γ chain (γ_c_) family, namely IL-2, IL-7, and IL-15, and an opposite effect driven by TGF-β1 that reduces DAP10 gene transcription^51^. Using a mouse model targeting DAP10 for ubiquitin-mediated degradation in NK cells, it has been shown that DAP10 transduces signals downstream of IL-15 receptor (IL-15R) and is required for IL-15 stimulation of NK cell survival, proliferation, and NKG2D-mediated cytotoxicity^50^. Of note, we found that HMBA-exposed NK cells with reduced DAP10 levels are unresponsive to IL-15 in terms of upmodulation of NKG2D and NKp44 receptor expression, NKG2D-mediated activation, and killing of transformed or infected cells. Our data strongly support the essential role of DAP10 in NK cells for the response to IL-15 stimulation and in coupling the NKG2D and IL-15R signaling pathways. Most importantly, the detrimental effects of HMBA observed *in vitro* in NK cells, even when activated by IL-15, suggest that HMBA administration may compromise NK cell-mediated elimination of tumor cells *in vivo*, a phenomenon that might have contributed to poor clinical outcome reported in HMBA-treated patients with myelo-dysplastic syndrome and acute leukemia^36,37^.

Results of the present study also show that the deleterious effects of HMBA on NK-cell phenotype and function may have negative implications for its employment in HIV-1 eradication strategies. The potential of HMBA in the reversal of HIV-1 latency has been recently reported, especially when combined with a second LRA belonging to a distinct functional category such as PRO^42,43^. By using a primary CD4^+^ T-cell based model of HIV-1 latency, we found that HMBA was devoid of the capacity to reactivate the virus when used alone or in combination with PRO, which is in contrast with previous reports^42,43^. Conflicting results are possibly due to differences in model systems and method of quantification, consisting in intracellular p24^+^ accumulation in primary T cells (this study) as opposed to HIV-1 Long terminal repeat (LTR)-driven transcription in T-cell lines^43^, or genomic viral RNA levels in culture supernatant of PBMCs from ART-treated patients^42^. Aligned with our data, a study based on latently infected primary T cells showed that treatment with PRO but not HMBA exposed these cells to recognition by HIV-1-specific CTLs^55^. Though devoid of LRA activity, we found that HMBA synergized with PRO at upregulating ULBP2 on those p24^+^ T cells harboring PRO-reactivated virus. These results are in agreement with the reported capacity of HMBA to prolong and potentiate NF-κB activation initiated by PRO via the suppression of the A20 deubiquitinase and, as a consequence, sustained degradation of the NF-κB inhibitor IκBα^43^. On the other hand, HMBA decreased NKG2D expression on NK cells in a dominant manner over PRO-mediated stimulation and damped NK cell-mediated clearance of p24^+^ T cell targets despite their higher ULBP2 expression in HMBA + PRO cultures. These results suggest that HMBA might be too weak to induce effective HIV-1 latency reversal, even when combined with a distinct LRA. In addition, the detrimental effects on the anti-viral responses of NK cells discourage the inclusion of HMBA in HIV-1 ‘shock-and-kill’ approaches, especially since results from initial clinical trials in ART patients indicate that NK cells have an important role, possibly superior to that of CTLs, in the context of viral eradication strategies^62–65^. Furthermore, HMBA may reduce NKG2D expression also on CD8^+^ T cells (our unpublished observations), which can compromise the cytotoxic activity of these cells upon NKG2D costimulation^66^, yet further studies are required to investigate this phenomenon.

Altogether, within the limitations of an *in vitro* study, results presented herein not only provide one possible explanation for the failure of HMBA in previous clinical trials, but also raise concerns for future therapeutic application of this drug. Indeed, by impairing expression of NKG2D in NK cells, HMBA hampers a crucial pathway in NK cell-mediated elimination of tumor and infected cells. Moreover, likely by impairing DAP10 expression, HMBA inhibits the capacity of NK cells to respond to IL-15, which is essential for NK cell development and function. Of note, IL-15 and, above all, IL-15 analogs with superior stability and efficacy are currently under investigation as immunotherapeutic agents for the treatment of solid and hematological tumors as well as for HIV-1 eradication^67^. Accumulating evidence shows that IL-15 agents have some efficacy as monotherapy, but they have a great potential when used in combination with chemotherapy agents, resulting in enhanced NK cell activity against tumor recurrence and metastasis in animal models^68^.

The unexpected inhibition exerted by HMBA on NK cell function overriding IL-15 stimulation disclosed in the present study emphasizes that novel hybrid polar agents and, more in general, anticancer drugs being taken forwards in clinical trials either alone or in combination, should be systematically tested for their impact on the effector function of NK cells and, conceivably, other cytotoxic cells.

## Materials and Method

### Cell lines and reagents

Primary NK and CD4^+^ T cells, FcγR^+^ P815, K562, Jurkat E6-1, CCRF-CEM (CEM), and MOLT-4 cells were maintained in RPMI 1640 medium supplemented with 10% fetal bovine serum, 0.2 mM L-glutamine, and 100 units/ml penicillin-streptomycin (all from Euroclone).

PBMCs were obtained by Ficoll separation of buffy coats from the donor bank of the Policlinico Tor Vergata (PTV; Rome, Italy). Use of buffy coats was approved by the PTV Ethical committee and written informed consent from all participants was obtained, in accordance with the Declaration of Helsinki.

Primary NK and CD4^+^ T cells were isolated from PBMCs by negative selection with cell-type specific EasySep CD4^+^ T-cell Enrichment Kit (Stem Cell Technologies) according to manufacturer’s protocol.

The purity (~95%) of isolated NK (CD3^−^CD56^+^CD16^−/+^) and CD4^+^ T cells (CD3^+^CD4^+^) was assessed by immunolabeling and FACS analysis.

For flow cytometry, isotype control IgG_1_ and IgG_2a_, either conjugated or not, (BD Pharmingen) and the following mouse monoclonal antibodies (mAbs) were used: CD3/AlexaFluor700 (UCHT1), CD56/PerCpCy5.5 (B159), CD16/BV510 (3G8), CD4/PerCp (L200), CD69/PE (FN50) from BD Pharmingen; CD56/PerCp (MEM-188) from Thermo Fisher Scientific; NKG2D(CD314)/PE (1D11), CD3/APC (UCHT1), CD16/APC-eFluor780 (CB16) from eBioscience; PVR (CD155; SKII.4), CD107a/FITC (H4A3), DNAM-1(CD226)/FITC (11A8), NKp30/PE (P30-15), NKp44/PE (P44-8), NKp46/PE-Cy7 (9E2) from BioLegend; p24/FITC (KC57) from Beckman Coulter; MICA (AMO1) from BamOmaB; MICB (MAB1599), ULBP1 (MAB1380), and ULBP2/5/6 (MAB1298) from R&D Systems. As a secondary antibody, Alexa647- or Alexa488-coniugated goat anti-mouse IgG (GAM) (Invitrogen) was used.

For western blotting we employed antibodies against NKG2D (clone 3.1.1.1; kind gift of Carsten Watzl, IfADo, Dortmund, Germany), DAP10 (clone H-2, sc-133173; Santa Cruz Biotechnology), and GAPDH (glyceraldehyde-3-phosphate dehydrogenase) (MAB374; Millipore), then horseradish peroxidase-conjugated secondary antibody (Cell Signaling).

The anti-NKG2D (149810; R&D Systems) mAb or isotype control IgG_1_ was used in the internalization assay, cytotoxicity, and redirected NK-cell lysis.

When indicated, cells were treated with 5 μM HMBA, 1 μM PRO, 10 μg/ml PHA (all from Sigma-Aldrich), 12.5 ng/ml IL-15 (Peprotech), and 29 nM CCL19 (R&D Systems).

### Flow cytometry

To assess viability, cells were stained with LIVE/DEAD fixable NEAR-IR dead cell stain kit according to manufacturer’s protocol (Life Technologies). To stain CD107a, cell cultures were supplemented with CD107a/FITC mAb (or control IgG_1_/FITC) for 6 h, with the addition of monensin (Golgi stop, diluted 1:1500; BD Pharmingen) and 10 μg/ml Brefeldin A (Sigma-Aldrich) after the first hour. The cell-surface and intracellular staining procedures were performed as described previously^14^. Immunolabeled cells resuspended in 1% paraformaldehyde (PFA) were acquired on a FACSCanto II (BD Biosciences) or Cytoflex (Beckman Coulter). Positive cell gating was set using fluorescence minus one control (FMO). Mean fluorescence intensity (MFI) was subtracted of the value obtained with isotype control antibody. Data analyses were performed using FlowJo software (TreeStar).

### Internalization assay

To measure the rate of NKG2D internalization, NK cells were collected after 18 h of culture with or without 5 mM HMBA, washed with cold PBS, reacted with anti-NKG2D mAb (0.05 μg/μl in PBS) for 30 min on ice, washed twice, and placed at 37 °C and 5% CO_2_. The endocytosis reaction was stopped after 5, 15, and 30 min of incubation by transferring an aliquot of cells on ice and adding NaN_3_ at a final 0.5% concentration. Next, the cells were stained with secondary antibody and analyzed by flow cytometry.

### Western blotting

NK cells were lysed as described previously^69^, then 20 μg of total cell lysate was separated on 10% SDS-PAGE and immunoblotted with primary then secondary antibodies. The protein-specific signals were detected with Pierce ECL substrate (Thermo Scientific) and quantified by densitometry.

### Real-time qPCR

Briefly, total RNA was extracted from NK cells treated or not with HMBA for 18 h using TRIzol (Life Technologies). Aliquots (1 μg) of total RNA were used to generate cDNA using random hexamers and the resulting cDNA (25 ng) was amplified in triplicate using the SensiFAST SYBR Green PCR master mix (all from Bioline). For each target (*NKG2D*, *DAP10*, and the *18 S rRNA* calibrator gene), forward (F) and reverse (R) primers were designed across exons to avoid amplification of genomic DNA, as validated in pilot assays: NKG2D(F), 5′-TCTCGACACAGCTGGGAGATG-3′; NKG2D(R), 5′-GACATCTTTGCTTTTGCCATCGTG-3′; DAP10(F), 5′-GGCACTTCAGGCTCTTGTTC-3′; DAP10(R), 5′-CCAGGATGAGAGGGTCAGAA-3′; 18 S rRNA(F), 5 -GAGGCCCTGTAATTGGAATGAG-3; 18 S rRNA(R), 5′-GCAGCAACTTTAATATACGCTATTGG-3′. The cycling conditions were 95 °C for 2 min, followed by 40 cycles of 95 °C for 5 sec and 60 °C for 30 sec. Real-time PCR was performed using Applied Biosystems 7300 Real-Time PCR System.

### Redirected antibody-dependent degranulation assay

PBMCs (effectors) cultivated for 18 h in medium alone (nt) or supplemented with 5 mM HMBA and/or 12,5 ng/ml IL-15 were seeded in plates in a 96-well plate (0.2 × 10^6^/well). FcγR^+^ P815 cells (targets) were loaded with anti-NKG2D mAb (5 μg/10^6^ cells) or equivalent amounts of IgG_1_ for 15 min at room temperature, then washed and plated with effectors at 1:2.5 E:T ratio (0.5 × 10^6^/well) in complete medium supplemented with anti-CD107a/FITC or IgG_1_/FITC and incubated at 37 °C for 6 h. After the first hour, monensin (Golgi stop, diluted 1:1500) and 10 μg/ml Brefeldin A were added to cultures. Finally cells were immunolabeled and analyzed by flow cytometry to measure the frequency of CD107a^+^ cells within gated NK cell population (CD3^+^CD56^+^CD16^+/−^).

### NK-cell cytotoxicity assays

Flow cytometry-based cytotoxicity assays were performed as described previously^14^, using K562 cells as targets and purified primary NK cells treated for 18 h with 5 μM HMBA and 12.5 ng/ml IL-15, either alone or in combination, or not treated (nt), as effectors. In addition, Jurkat, CEM, or MOLT-4 cells that have been cultivated for 18 h with or without HMBA were used as targets of NK cells pre-treated for 18 h with IL-15 alone or together with HMBA. Briefly, 5,6-carboxyfluorescein diacetate succinimidyl ester (CFSE; Sigma-Aldrich)-labeled NK cells were co-cultivated with target cells at different effector-to-target cell (E:T) ratios for 4 h. When indicated, NK cells were treated with anti-NKG2D blocking antibody (1 μg/10^6^ cells) or control IgG_1_ for 15 minutes prior to incubation with target cells. Next, cells were labeled with 7-aminoactinomycin D (7-AAD; Sigma-Aldrich), fixed and acquired by FACS. The percentage of specific lysis of target cells (gated as CFSE^−^) was calculated as follows: 100 × (%7-AAD^+^ target cells in sample − basal %7-AAD^+^ target cells)/(100 − basal %7-AAD^+^ target cells). For K562 targets, specific lysis derived from three E:T ratios was conversed to lytic units (LU), defined as the number of effector cells required to lyse 20% of 2 × 10^5^ target cells^70^, and results were expressed as the number of LU contained in 10^7^ NK cells.

### Establishment and reactivation of latently infected CD4^+^ T cells

Primary CD4^+^ T cells cultures infected with HIV-1 were established and then reactivated as previously described with minor modifications^71^. Briefly, purified CD4^+^ T cells were cultivated with 29 nM CCL19 (R&D Systems) for 1–3 days. After this period, the quiescent state of the cells was verified by excluding the presence of CD69^+^, CD25^+^, and HLA-DR^+^ cells by FACS analysis. Then, cells were infected by spinoculation with 300 ng p24/10^6^ cells of NL4-3 HIV-1 (NIH AIDS Reagent Program) pseudotyped with vesicular stomatitis virus glycoprotein (VSV-G), washed, and placed back in culture in complete medium without chemokine or cytokines. To activate the virus, latently infected CD4^+^ T cells were exposed to 5 μM HMBA and 1 μΜ PRO, either alone or in combination, to 10 μg/ml PHA, or not stimulated (ns) at day 3 post-infection. Finally, at 72 h post-stimulation, cells were harvested and analyzed by FACS for intracellular p24 accumulation and cell-surface NKG2DLs expression.

### NK cell-mediated killing of reactivated HIV-1-infected cells

Primary HIV-1-infected CD4^+^ T cells (targets) reactivated with 1 μΜ PRO alone or together with 5 mM HMBA or not treated (nt), were collected 54 h post-stimulation and further cultivated for 18 h in the same treatment conditions (nt, PRO, PRO + HMBA) either alone or together with NK cells (effectors) purified from a cryopreserved aliquot of PBMCs of the same donor at an E:T ratio of 1:1. Cells were then fixed/permeabilized and stained for p24 and CD3. Finally, 7,000 target cells (gated as CD3^+^) were acquired by FACS. The percent NK cell-mediated killing of p24^+^ cells was calculated with the following formula: 100 × (%p24^+^ cells in targets − %p24^+^ cells in targets with effectors)/(%p24^+^ cells in targets).

### Statistical analysis

All experiments have been performed independently at least three times. GraphPad Prism 6.0 software was used to perform all statistical analyses. A value of *P* < 0.05 was considered statistically significant.

## Supplementary information

Dataset 1

## Data Availability

The datasets generated during the current study are available from the corresponding author on reasonable request.

## References

[1] Lanier LL (2005). NK cell recognition. Annu. Rev. Immunol..

[2] Bachanova V, Miller JS (2014). NK cells in therapy of cancer. Crit. Rev. Oncog..

[3] Rezvani K, Rouce RH (2015). The Application of Natural Killer Cell Immunotherapy for the Treatment of Cancer. Front. Immunol..

[4] Koehl U (2015). Advances in clinical NK cell studies: Donor selection, manufacturing and quality control. Oncoimmunology.

[5] Veluchamy JP (2017). The Rise of Allogeneic Natural Killer Cells As a Platform for Cancer Immunotherapy: Recent Innovations and Future Developments. Front. Immunol..

[6] Krieg S, Ullrich E (2013). Novel immune modulators used in hematology: impact on NK cells. Front. Immunol..

[7] Cifaldi L, Locatelli F, Marasco E, Moretta L, Pistoia V (2017). Boosting Natural Killer Cell-Based Immunotherapy with Anticancer Drugs: a Perspective. Trends Mol. Med..

[8] Raulet DH (2003). Roles of the NKG2D immunoreceptor and its ligands. Nat. Rev. Immunol..

[9] Raulet DH, Gasser S, Gowen BG, Deng W, Jung H (2013). Regulation of ligands for the NKG2D activating receptor. Annu. Rev. Immunol..

[10] Wu J (1999). An activating immunoreceptor complex formed by NKG2D and DAP10. Science.

[11] Upshaw JL (2006). NKG2D-mediated signaling requires a DAP10-bound Grb2-Vav1 intermediate and phosphatidylinositol-3-kinase in human natural killer cells. Nat. Immunol..

[12] Lanier LL (2015). NKG2D Receptor and Its Ligands in Host Defense. Cancer. Immunol. Res..

[13] Fogli M (2008). Lysis of endogenously infected CD4+ T cell blasts by rIL-2 activated autologous natural killer cells from HIV-infected viremic individuals. PLoS Pathog..

[14] Desimio MG, Giuliani E, Doria M (2017). The histone deacetylase inhibitor SAHA simultaneously reactivates HIV-1 from latency and up-regulates NKG2D ligands sensitizing for natural killer cell cytotoxicity. Virology.

[15] Deeks SG (2012). HIV: Shock and kill. Nature.

[16] Margolis DM, Archin NM (2017). Proviral Latency, Persistent Human Immunodeficiency Virus Infection, and the Development of Latency Reversing Agents. J. Infect. Dis..

[17] Mbonye U, Karn J (2017). The Molecular Basis for Human Immunodeficiency Virus Latency. Annu. Rev. Virol..

[18] Margolis DM, Garcia JV, Hazuda DJ, Haynes BF (2016). Latency reversal and viral clearance to cure HIV-1. Science.

[19] Rasmussen TA, Lewin SR (2016). Shocking HIV out of hiding: where are we with clinical trials of latency reversing agents?. Curr. Opin. HIV. AIDS..

[20] Thorlund, K., Horwitz, M. S., Fife, B. T., Lester, R. & Cameron, D. W. Landscape review of current HIV ‘kick and kill’ cure research - some kicking, not enough killing. *BMC Infect. Dis*. **17**, 595-017-2683-3 (2017). 10.1186/s12879-017-2683-3 PMC5576299 28851294

[21] Chretien AS (2014). Cancer-Induced Alterations of NK-Mediated Target Recognition: Current and Investigational Pharmacological Strategies Aiming at Restoring NK-Mediated Anti-Tumor Activity. Front. Immunol..

[22] Desimio MG, Giuliani E, Ferraro AS, Adorno G, Doria M (2018). *In Vitro* Exposure to Prostratin but Not Bryostatin-1 Improves Natural Killer Cell Functions Including Killing of CD4^+^ T Cells Harboring Reactivated Human Immunodeficiency Virus. Front. Immunol..

[23] Garrido C (2018). Interleukin-15-Stimulated Natural Killer Cells Clear HIV-1-Infected Cells following Latency Reversal *Ex Vivo*. J. Virol..

[24] Ogbomo H, Michaelis M, Kreuter J, Doerr HW, Cinatl J (2007). Histone deacetylase inhibitors suppress natural killer cell cytolytic activity. FEBS Lett..

[25] Rossi LE (2012). Histone deacetylase inhibitors impair NK cell viability and effector functions through inhibition of activation and receptor expression. J. Leukoc. Biol..

[26] Clutton G (2016). The differential short- and long-term effects of HIV-1 latency-reversing agents on T cell function. Sci. Rep..

[27] Garrido C (2016). HIV Latency-Reversing Agents Have Diverse Effects on Natural Killer Cell Function. Front. Immunol..

[28] Pace M (2016). Histone Deacetylase Inhibitors Enhance CD4 T Cell Susceptibility to NK Cell Killing but Reduce NK Cell Function. PLoS Pathog..

[29] Walker-Sperling VE, Pohlmeyer CW, Tarwater PM, Blankson JN (2016). The Effect of Latency Reversal Agents on Primary CD8^+^ T Cells: Implications for Shock and Kill Strategies for Human Immunodeficiency Virus Eradication. EBioMedicine.

[30] Haces A, Breitman TR, Driscoll JS (1987). Chemical differentiating agents. Differentiation of HL-60 cells by hexamethylenebis[acetamide] analogues. J. Med. Chem..

[31] Marks PA, Richon VM, Kiyokawa H, Rifkind RA (1994). Inducing differentiation of transformed cells with hybrid polar compounds: a cell cycle-dependent process. Proc. Natl. Acad. Sci. USA.

[32] Sparatore B (1995). Changes in calcium influx affect the differentiation of murine erythroleukaemia cells. Biochem. J..

[33] Richon VM (1997). Two cytodifferentiation agent-induced pathways, differentiation and apoptosis, are distinguished by the expression of human papillomavirus 16 E7 in human bladder carcinoma cells. Cancer Res..

[34] Mallia CM (1999). Protein kinase calpha is an effector of hexamethylene bisacetamide-induced differentiation of Friend erythroleukemia cells. Exp. Cell Res..

[35] Cecchinato V (2008). Hexamethylene bisacetamide inhibits malignant phenotype in T-ALL cell lines. Leuk. Res..

[36] Andreeff M (1992). Hexamethylene bisacetamide in myelodysplastic syndrome and acute myelogenous leukemia: a phase II clinical trial with a differentiation-inducing agent. Blood.

[37] Rowinsky EK (1992). Hexamethylene bisacetamide in myelodysplastic syndrome: effect of five-day exposure to maximal therapeutic concentrations. Leukemia.

[38] Contreras X, Barboric M, Lenasi T, Peterlin BM (2007). HMBA releases P-TEFb from HEXIM1 and 7SK snRNA via PI3K/Akt and activates HIV transcription. PLoS Pathog..

[39] Choudhary SK, Archin NM, Margolis DM (2008). Hexamethylbisacetamide and disruption of human immunodeficiency virus type 1 latency in CD4^+^ T cells. J. Infect. Dis..

[40] Chen R (2008). PP2B and PP1alpha cooperatively disrupt 7SK snRNP to release P-TEFb for transcription in response to Ca2+ signaling. Genes Dev..

[41] Ai N (2011). Signal-induced Brd4 release from chromatin is essential for its role transition from chromatin targeting to transcriptional regulation. Nucleic Acids Res..

[42] Darcis G (2015). An In-Depth Comparison of Latency-Reversing Agent Combinations in Various *In Vitro* and *Ex Vivo* HIV-1 Latency Models Identified Bryostatin-1 + JQ1 and Ingenol-B + JQ1 to Potently Reactivate Viral Gene Expression. PLoS Pathog..

[43] Chen D (2016). HMBA Enhances Prostratin-Induced Activation of Latent HIV-1 via Suppressing the Expression of Negative Feedback Regulator A20/TNFAIP3 in NF-κB Signaling. Biomed. Res. Int..

[44] Richon VM (1996). Second generation hybrid polar compounds are potent inducers of transformed cell differentiation. Proc. Natl. Acad. Sci. USA.

[45] Bottino C (2003). Identification of PVR (CD155) and Nectin-2 (CD112) as cell surface ligands for the human DNAM-1 (CD226) activating molecule. J. Exp. Med..

[46] Salih HR (2003). Functional expression and release of ligands for the activating immunoreceptor NKG2D in leukemia. Blood.

[47] Hilpert J (2012). Comprehensive analysis of NKG2D ligand expression and release in leukemia: implications for NKG2D-mediated NK cell responses. J. Immunol..

[48] Klichko V, Archin N, Kaur R, Lehrman G, Margolis D (2006). Hexamethylbisacetamide remodels the human immunodeficiency virus type 1 (HIV-1) promoter and induces Tat-independent HIV-1 expression but blunts cell activation. J. Virol..

[49] Molfetta R (2016). Regulation of NKG2D Expression and Signaling by Endocytosis. Trends Immunol..

[50] Horng T, Bezbradica JS, Medzhitov R (2007). NKG2D signaling is coupled to the interleukin 15 receptor signaling pathway. Nat. Immunol..

[51] Park YP (2011). Complex regulation of human NKG2D-DAP10 cell surface expression: opposing roles of the gammac cytokines and TGF-β1. Blood.

[52] Pende D (2002). Major histocompatibility complex class I-related chain A and UL16-binding protein expression on tumor cell lines of different histotypes: analysis of tumor susceptibility to NKG2D-dependent natural killer cell cytotoxicity. Cancer Res..

[53] Cerboni C (2007). Human immunodeficiency virus 1 Nef protein downmodulates the ligands of the activating receptor NKG2D and inhibits natural killer cell-mediated cytotoxicity. J. Gen. Virol..

[54] Vlach J, Pitha PM (1993). Hexamethylene bisacetamide activates the human immunodeficiency virus type 1 provirus by an NF-kappa B-independent mechanism. J. Gen. Virol..

[55] Jones RB (2016). A Subset of Latency-Reversing Agents Expose HIV-Infected Resting CD4^+^ T-Cells to Recognition by Cytotoxic T-Lymphocytes. PLoS Pathog..

[56] Skov S (2005). Cancer cells become susceptible to natural killer cell killing after exposure to histone deacetylase inhibitors due to glycogen synthase kinase-3-dependent expression of MHC class I-related chain A and B. Cancer Res..

[57] Vales-Gomez M, Chisholm SE, Cassady-Cain RL, Roda-Navarro P, Reyburn HT (2008). Selective induction of expression of a ligand for the NKG2D receptor by proteasome inhibitors. Cancer Res..

[58] Luis Espinoza J, Takami A, Trung LQ, Nakao S (2013). Ataxia-telangiectasia mutated kinase-mediated upregulation of NKG2D ligands on leukemia cells by resveratrol results in enhanced natural killer cell susceptibility. Cancer. Sci..

[59] Satwani P (2014). Upregulation of NKG2D ligands in acute lymphoblastic leukemia and non-Hodgkin lymphoma cells by romidepsin and enhanced *in vitro* and *in vivo* natural killer cell cytotoxicity. Cytotherapy.

[60] Uhlenbrock F (2014). The NKG2D ligand ULBP2 is specifically regulated through an invariant chain-dependent endosomal pathway. J. Immunol..

[61] Xu J (2012). Transcriptional pausing controls a rapid antiviral innate immune response in Drosophila. Cell. Host Microbe.

[62] Olesen R (2015). Innate Immune Activity Correlates with CD4 T Cell-Associated HIV-1 DNA Decline during Latency-Reversing Treatment with Panobinostat. J. Virol..

[63] Garrido, C. *et al*. Clinical administration of Vorinostat increases NK cell capacity to produce IFN-γamma. Abstr 355 presented at: Conference on Retroviruses and Opportunistic Infections; Boston, MA, USA (2016).

[64] Offersen R (2016). A Novel Toll-Like Receptor 9 Agonist, MGN1703, Enhances HIV-1 Transcription and NK Cell-Mediated Inhibition of HIV-1-Infected Autologous CD4^+^ T Cells. J. Virol..

[65] Vibholm L (2017). Short-Course Toll-Like Receptor 9 Agonist Treatment Impacts Innate Immunity and Plasma Viremia in Individuals With Human Immunodeficiency Virus Infection. Clin. Infect. Dis..

[66] Giuliani E (2015). Expression and Function of NKG2D Is Impaired in CD8^+^ T Cells of Chronically HIV-1-Infected Patients Without ART. J. Acquir. Immune Defic. Syndr..

[67] Guo Y, Luan L, Patil NK, Sherwood ER (2017). Immunobiology of the IL-15/IL-15Rα complex as an antitumor and antiviral agent. Cytokine Growth Factor Rev..

[68] Robinson TO, Schluns KS (2017). The potential and promise of IL-15 in immuno-oncogenic therapies. Immunol. Lett..

[69] Neri F, Giolo G, Potesta M, Petrini S, Doria M (2011). The HIV-1 Nef protein has a dual role in T cell receptor signaling in infected CD4^+^ T lymphocytes. Virology.

[70] Bryant J, Day R, Whiteside TL, Herberman RB (1992). Calculation of lytic units for the expression of cell-mediated cytotoxicity. J. Immunol. Methods.

[71] Saleh S (2011). Expression and reactivation of HIV in a chemokine induced model of HIV latency in primary resting CD4^+^ T cells. Retrovirology.

