# Supplementary material for: Hexamethylene bisacetamide impairs NK cell-mediated clearance of acute T lymphoblastic leukemia cells and HIV-1-infected T cells that exit viral latency
Source: Sci Rep. 2019 Mar 13;9:4373. doi: 10.1038/s41598-019-40760-x (PMC6416400; doi:10.1038/s41598-019-40760-x)
Supplement: Supplementary file 1 — Dataset 1 [file 41598_2019_40760_MOESM1_ESM.pdf]

Supplementary Figure S1

Hexamethylene bisacetamide impairs NK cell-mediated clearance of acute T lymphoblastic leukemia cells and HIV-1-infected T cells that exit viral latency

Erica Giuliani, Maria Giovanna Desimio, and Margherita Doria

Original blots for figure 3D

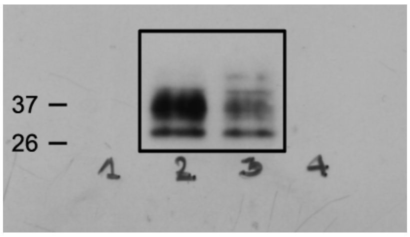

Probed with anti-NKG2D mAb

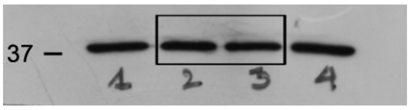

Same filter re-probed with anti-GAPDH mAb

Original blots for figure 3E

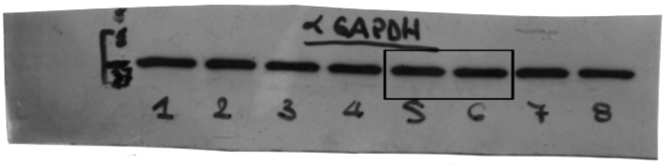

Top filter: probed with anti-GAPDH mAb

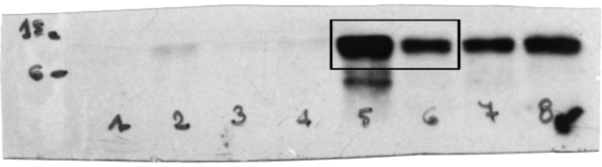

Bottom: probed with anti-DAP10 mAb

Supplementary Figure S1. Uncropped images of the western blots. The original images of the western blots depicted in figures 3D and 3E are shown. The boxed areas indicate the regions dysplayed in the manuscript
